# Exploring the GLP-1–GLP-1R axis in porcine pancreas and gastrointestinal tract in vivo by ex vivo autoradiography

**DOI:** 10.1136/bmjdrc-2020-002083

**Published:** 2021-04-26

**Authors:** Elin Manell, Emmi Puuvuori, Anna Svensson, Irina Velikyan, Gry Hulsart-Billström, Patricia Hedenqvist, Jens Juul Holst, Marianne Jensen Waern, Olof Eriksson

**Affiliations:** 1 Department of Clinical Sciences, Swedish University of Agricultural Sciences, Uppsala, Sweden; 2 Science for Life Laboratory, Department of Medicinal Chemistry, Uppsala University, Uppsala, Sweden; 3 NNF Centre for Basic Metabolic Research and Department of Biomedical Sciences, University of Copenhagen, Copenhagen, Denmark

**Keywords:** incretins, glucagon-like peptide 1, receptors, gastrointestinal hormone, glucose tolerance test

## Abstract

**Introduction:**

Glucagon-like peptide-1 (GLP-1) increases insulin secretion from pancreatic beta-cells and GLP-1 receptor (GLP-1R) agonists are widely used as treatment for type 2 diabetes mellitus. Studying occupancy of the GLP-1R in various tissues is challenging due to lack of quantitative, repeatable assessments of GLP-1R density. The present study aimed to describe the quantitative distribution of GLP-1Rs and occupancy by endogenous GLP-1 during oral glucose tolerance test (OGTT) in pigs, a species that is used in biomedical research to model humans.

**Research design and methods:**

GLP-1R distribution and occupancy were measured in pancreas and gastrointestinal tract by ex vivo autoradiography using the GLP-1R-specific radioligand ^177^Lu-exendin-4 in two groups of pigs, control or bottle-fed an oral glucose load. Positron emission tomography (PET) data from pigs injected with ^68^Ga-exendin-4 in a previous study were used to retrieve data on biodistribution of GLP-1R in the gastrointestinal tract.

**Results:**

High homogenous uptake of ^177^Lu-exendin-4 was found in pancreas, and even higher uptake in areas of duodenum. Low uptake of ^177^Lu-exendin-4 was found in stomach, jejunum, ileum and colon. During OGTT, there was no increase in plasma GLP-1 concentrations and occupancy of GLP-1Rs was low. The ex vivo autoradiography results were highly consistent with to the biodistribution of ^68^Ga-exendin-4 in pigs scanned by PET.

**Conclusion:**

We identified areas with similarities as well as important differences regarding GLP-1R distribution and occupancy in pigs compared with humans. First, there was strong ligand binding in the exocrine pancreas in islets. Second, GLP-1 secretion during OGTT is minimal and GLP-1 might not be an important incretin in pigs under physiological conditions. These findings offer new insights on the relevance of porcine diabetes models.

**Significance of this study**

**What is already known about this subject?**

- In humans, oral intake of glucose stimulates glucagon-like peptide-1 (GLP-1) release from intestinal L-cells.
- Glucagon-like peptide-1 receptor (GLP-1R) locations have been identified with in vitro methods in different parts of the body including pancreas and gastrointestinal tract, and GLP-1R densities have been estimated to be larger in the islets compared with exocrine tissue in human pancreas.

**What are the new findings?**

- GLP-1R distribution in pancreas and gastrointestinal tract was quantified in vivo by ex vivo autoradiography using the GLP-1R-specific radioligand ^177^Lu-exendin-4 in pigs.
- There are areas in duodenum with higher exendin-4 uptake than in pancreas.
- In pancreas, exendin-4 uptake was equally distributed across the entire autoradiography image and not predominantly found in the islets.
- Results from ex vivo autoradiography were highly consistent with the biodistribution of ^68^Ga-exendin-4 in pigs scanned by positron emission tomography (PET), suggesting that GLP-1R distribution in the gastrointestinal tract can be studied in vivo by PET scanner.
- GLP-1 secretion in response to an oral glucose load of 2.5 g/kg body weight is minimal in pigs, and GLP-1R occupancy by endogenous GLP-1 is low.

**How might these results change the focus of research or clinical practice?**

- This study identified important differences in GLP-1 regulation and secretion between pigs and humans, which must be kept in mind when using pigs as animal model for diabetes.
- Studies investigating GLP-1R target distribution and occupancy, also in the gastrointestinal tract, can be safely and accurately performed in vivo using ^68^Ga-exendin-4, both in pigs and humans.

## Introduction

Diabetes mellitus (DM) is a global disease with >420 million people affected.^1^ Although several pharmaceuticals with glucose-lowering effects have been developed to alleviate clinical signs of disease, many patients with DM still suffer from long-term complications such as cardiovascular disease, renal disease and vision impairment due to poor glycemic control.^1^

Glucagon-like peptide-1 (GLP-1) secretion from intestinal L-cells after nutrient ingestion increases insulin release from pancreatic beta-cells.^2 3^ Although the effect of physiological levels of GLP-1 is impaired in type 2 DM,^4 5^ the GLP-1 receptor agonists (GLP-1RAs) are still active and are being widely used as treatment for type 2 DM. GLP-1RAs have also recently been shown to improve glycemic control in combination with insulin treatment in patients with type 1 DM.^6^ Despite decades of research, the physiological mechanisms of GLP-1 and its role in the pathophysiology of DM are not fully understood. GLP-1 binds to the G-protein coupled glucagon-like peptide 1 receptor (GLP-1R)^7^ and many attempts have been made to study the localisation of GLP-1Rs in various tissues. However, due to limitations in specificity of most anti-GLP-1R antibodies, the reliability of the immunohistochemical (IHC) studies has been questioned.^8^ Furthermore, possible species differences between rodents and humans limits the use of rodents for this research question.^9^ Within the gastrointestinal tract, GLP-1R has been found in non-human primate stomach, duodenum, jejunum, ileum and colon by IHC staining of tissues with an extensively validated antibody for primate tissues.^10^ However, detailed quantifications of receptors are not possible with IHC methods and it is also desirable to develop experimental research models that do not require the use of non-human primates.^11^ Furthermore, the lack of quantitative, repeatable assessments of GLP-1R density means that it is difficult to assess target engagement or occupancy, for example, by pharmaceutical GLP-1RAs. The required proportion of engaged receptors by either endogenous GLP-1 peptide or drug candidates for a significant physiological effect, is thus unknown. This hampers the understanding of GLP-1 mode of action, treatment dose/ effect as well as optimal dosing regimen in relation to adverse events.

Pigs are commonly used as a large animal model in diabetes research since they are anatomically and physiologically very similar to humans,^12^ and oral glucose tolerance test (OGTT) can be carried out in the same way with pigs as with humans.^13^ The aim of the present experiment was, with the aid of ex vivo autoradiography and radioactively labelled exendin-4, to quantitatively describe the distribution of GLP-1Rs in pancreas and gastrointestinal tract and to assess GLP-1R occupancy by endogenous GLP-1 in vivo during OGTT in pigs.

## Research design and methods

### Animals

Sixteen high-health pigs (Yorkshire×Hampshire, both sexes) were obtained from the University herd (Swedish Livestock Research Centre, Lövsta, Sweden) in two rounds. Ten pigs, aged 5 weeks (experiment 1) and six pigs, aged 7 weeks (experiment 2) were used. At the time of experiment, the pigs weighed 22±2 kg in experiment 1 and 55±3 kg in experiment 2. The animals were housed at the Department of Clinical Sciences in individual pens measuring 3 m^2^, within sight and sound of one another. Straw and wood shavings were used as bedding. A 10:14 hour light/dark schedule (lights on at 06:00 hours) was applied and an infrared lamp (24 hours) was provided in each pen. The room temperature was set at 16°C–18°C. The pigs were fed commercial finisher diet (SOLO 330, Lantmännen, Sweden) two times per day (07:00 and 15:00 hours), the amount depending on body weight according to the Swedish University of Agricultural Sciences regimen for growing pigs.^14^ Water was provided ad libitum. During a 2-week acclimatisation period, each pig was handled 15 min/day to become accustomed to all the people participating in the study, and trained to bottle-feed glucose dissolved in water as previously described.^13^ Bottle-feeding training was continued until the experiment. The pigs were also trained to step onto an electronic scale and were clinically examined by experienced veterinarians. Details of blood analyses prior to inclusion in experiment and indwelling catheter surgery are found in online supplemental material 1.

10.1136/bmjdrc-2020-002083.supp1 Supplementary data

### Experiment 1

The preparation of [^177^Lu]Lu-DOTA-exendin-4-Nle14 (^177^Lu-exendin-4) is described in online supplemental material 1. All 10 pigs were injected intravenously with ^177^Lu-exendin-4, 1 MBq/kg body weight (BW). The administered activity was corrected for the remnants of radioactivity in the injection syringe. The injected radioactivity amount corresponded to a peptide mass dose of 0.03–0.05 µg/kg DOTA-VS-exendin-4 in all pigs. Blood samples were collected in lithium heparin tubes for measurement of radioactivity concentration in plasma and whole blood at 10, 20, 30, 40, 50 and 60 min after ^177^Lu-exendin-4 injection. Radioactivity concentration was measured in a gamma counter (Uppsala Imanet, Uppsala, Sweden).

Four pigs were bottle-fed an oral glucose load, 2.5 g glucose/kg BW dissolved in 2 mL water/g glucose (Glukos APL Pulver till oral lösning, APL, Stockholm, Sweden), 10 min before ^177^Lu-exendin-4 injection; the glucose solution had to be consumed within 5 min. Blood samples were collected in EDTA tubes for measurement of blood glucose concentrations and plasma concentrations of insulin and GLP-1 before and 15, 30, 45 and 70 min after glucose intake. Blood samples were collected from control animals at corresponding times after ^177^Lu-exendin-4 injection. All pigs were euthanized 60 min after ^177^Lu-exendin-4 injection.

### Experiment 2

After results from experiment 1 had been analyzed, experiment 2 was carried out in order to investigate if GLP-1R occupancy with endogenous GLP-1 could be detected in all intestinal segments with an earlier end point of 30 min. All six pigs were injected intravenously with ^177^Lu-exendin-4, 0.5 MBq/kg BW. The administered activity was corrected for the remnants of radioactivity in the injection syringe. The injected radioactivity amount corresponded to a peptide mass dose of 0.03–0.05 µg/kg DOTA-VS-exendin-4 in all pigs. Blood samples were collected in lithium heparin tubes for measurement of radioactivity concentration in plasma and whole blood at 10, 20, and 30 min after ^177^Lu-exendin-4 injection, as described above.

Three pigs underwent OGTT as described in experiment 1. Immediately following oral glucose intake, ^177^Lu-exendin-4 was injected intravenously. Blood samples were collected in EDTA tubes for measurement of blood glucose concentrations and plasma concentrations of insulin before and 10, 20 and 30 min after glucose intake. Blood samples were collected from control animals at corresponding times after ^177^Lu-exendin-4 injection. All pigs were euthanized 30 min after ^177^Lu-exendin-4 injection.

### Ex vivo autoradiography

The pigs were euthanized with intravenous injection of pentobarbital sodium (Pentobarbital vet. 100 mg/mL, Apoteksbolaget, Sweden). Immediately after euthanasia, tissue biopsies were collected from pancreas, stomach, proximal duodenum (5 cm from pyloric orifice), jejunum (50 cm from pyloric orifice), distal ileum (5 cm from ileocecal valve), colon and spleen (negative control) and frozen in liquid nitrogen. Samples were kept in −80°C until sectioned. Biopsies were embedded in OCT Cryomount (Histolab, Sweden) and processed into seven serial sections, no 2, 3, 5 and 6 (20 µm) for autoradiography and no 1, 4 and 7 (12 µm) for H&E staining for histology. In experiment 1, four slides were prepared for autoradiography, and results from each pig averaged. In experiment 2, one slide was prepared for autoradiography. The tissue slides were exposed against digital phosphorimager screens, together with known references of ^177^Lu, cross-calibrated against a gamma counter to enable quantification of the autoradiograms. The phosphorimaging plates were then developed by a Typhoon autoradiography reader (GE Healthcare, Chicago, Illinois, USA).

### Autoradiogram analysis

ImageJ (NIH) was used to analyze the autoradiography images. Segmentations were delineated on all tissues, and for duodenum also on internal subregions with clearly elevated binding. The references were similarly segmented. The binding in each tissue (expressed as counts/mm^2^) were corrected by the background of the plate as assessed by separate rectangular segmentations. All radioactivity measurements values (injected ^177^Lu-exendin-4, autoradiography binding and references measured in gamma counter) were decay-corrected to the end of synthesis of ^177^Lu-exendin-4 for each experiment, to enable direct quantification. The known reference (Bq/counts) was used to convert autoradiography binding values in counts/mm^2^ to Bq/mm^2^. The known thickness of the sections (20 µm) was used to convert the binding values to Bq/mm^3^. Finally, the specific radioactivity (GBq/μmol) of each batch ^177^Lu-exendin-4 at the end of synthesis was used to convert the binding in each tissue from Bq/mm^3^ to fmol/ mm^3^. The binding of ^177^Lu-exendin-4 in all slides of the respective tissue in each animal was averaged for the final reported value.

### Blood analyses

Blood glucose concentrations were measured with test strips (Accu-Chek, Roche Diagnostics, Basel, Switzerland; validated for porcine blood at the Department of Clinical Chemistry, SLU). Plasma insulin concentrations were determined by Porcine Insulin ELISA (10-1200-01, Mercodia, Uppsala, Sweden). All samples were run in duplicates with coefficient of variation (CV) <10%, except one sample with CV 13.9%. Interassay variations were 4.7%, 5.9% and 1.7% for 55.4, 17.6 and 5.04 mU/L standards (Animal Insulin Control, 10-1221-01, Mercodia, Uppsala, Sweden), respectively. Results were converted from mU/L to pmol/L for porcine insulin by factor 6, as recommended by the manufacturer. GLP-1 concentrations in plasma were measured by radioimmunoassays after extraction of plasma with 70% ethanol (vol/vol, final concentration). Carboxy-terminal GLP-1 immunoreactivity was determined using antiserum 89390,^15^ which has an absolute requirement for the intact amidated carboxy-terminus of GLP-1(7-36)amide and cross-reacts <0.01% with carboxy-terminally truncated fragments and 89% with GLP-1(9-36)amide, the primary metabolite of dipeptidyl-peptidase IV-mediated degradation. The sum of the two components (total GLP-1 concentration) reflects the rate of secretion of the L-cell. Sensitivity was <1 pmol/L, and intra-assay CV <5%.

### Retrospective analysis of exendin-4 positron emission tomography/CT in pigs

[^68^Ga]-DO3A-VS-Cys^40^-exendin-4 (^68^Ga-exendin-4) was previously used to assess the GLP-1R density in pancreas of healthy pigs in vivo, by positron emission tomography (PET)/CT.^16^ In the present study, we performed a retrospective qualitative visual assessment of the in vivo distribution of GLP-1Rs in the gastrointestinal tract as assessed by PET, to compare with the ex vivo binding in tissues as determined by ^177^Lu-exendin-4. Relevant tissues (pancreas, stomach, duodenum, small intestine, large intestine/colon and spleen) were identified on co-registered PET and CT images by the software PMOD V.3.7 (PMOD Technologies, Zürich, Switzerland). All images were normalized to standardized uptake value=6 to enable direct comparison.

### Statistical analyses

Values are presented as mean±SD. Comparisons between groups (control vs OGTT in experiment 1 and 2 separately) were made by unpaired t-test, and comparisons within groups (control pancreas vs duodenum) were made by paired t-test. All t-tests were run by GraphPad Prism V.8 (San Diego, California, USA). To be able to include animals from both experiments 1 and 2 in the analysis of intestinal segments with low ^177^Lu-exendin-4 uptake (spleen vs stomach, jejunum, ileum and colon, respectively) in control pigs, a single measures parametric analysis analysis of variance, blocked for experiment to account for differences in background signal, with Dunnett post hoc test was used, run by InVivoStat V.4.0.1 (www.invivostat.co.uk). Throughout the study, p values <0.05 were considered statistically significant.

### Data and resource availability

The datasets and resources generated during and/or analyzed during the current study are available from the corresponding author on reasonable request.

## Results

### Distribution of GLP-1Rs

**Figure 1:**
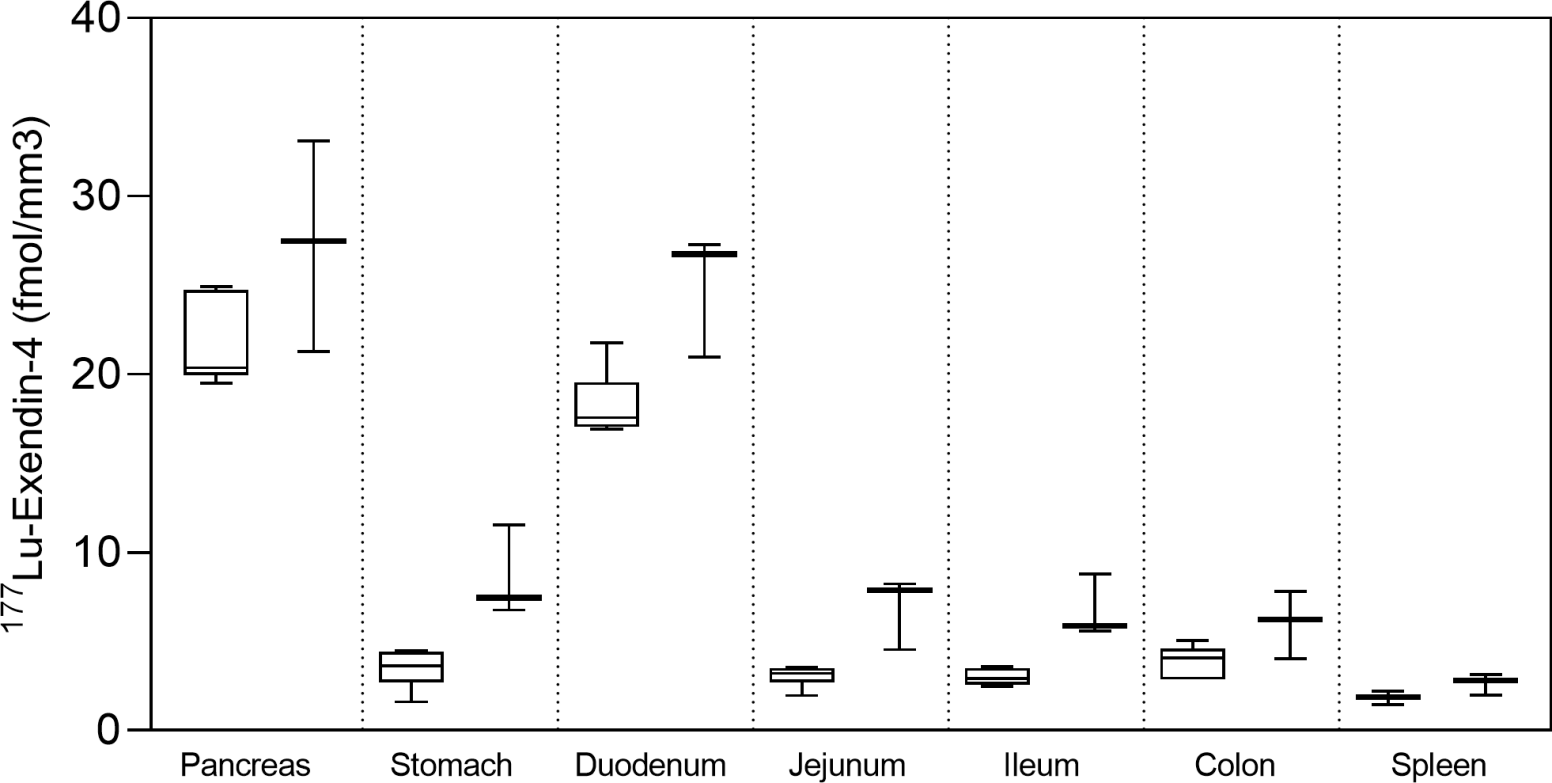
^177^Lu-exendin-4 in pancreas, stomach, duodenum, jejunum, ileum, colon and spleen measured by ex vivo autoradiography in control pigs in experiment 1 (left, n=6) and experiment 2 (right, n=3). Values are presented in box plots with first quartile, median and third quartile. Whiskers indicate min to max.

**Figure 2:**
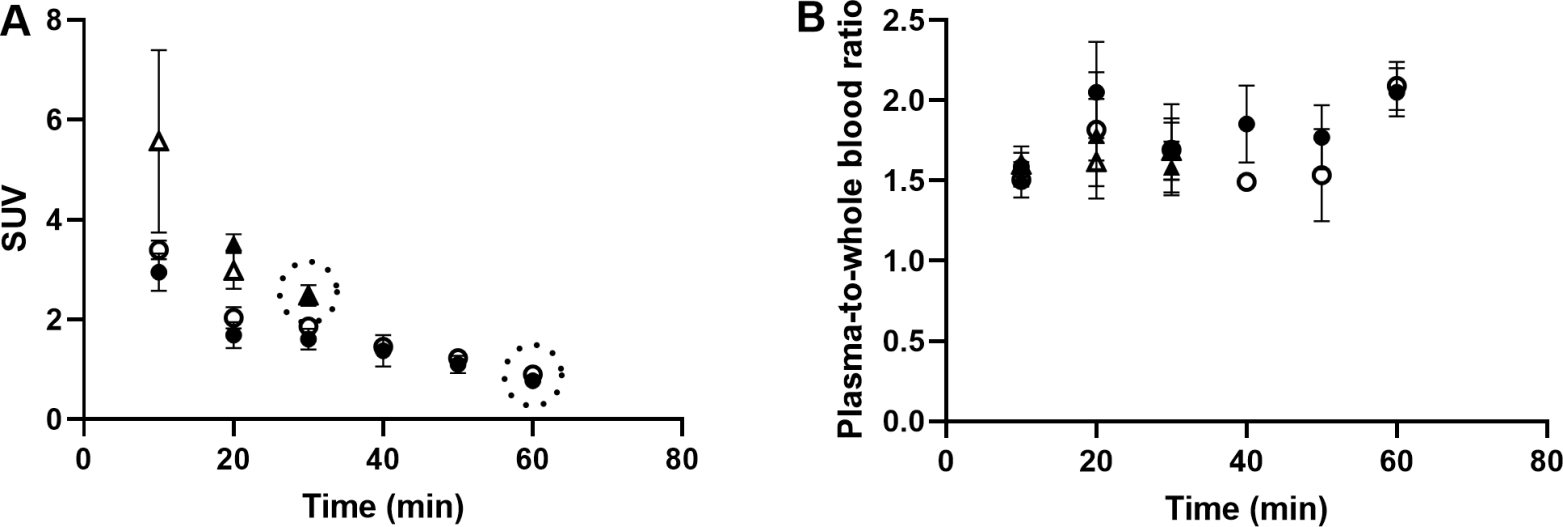
^177^Lu-exendin-4 in blood (A) and plasma (B) in pigs in experiment 1, control group (n=6) filled circles, oral glucose tolerance test (OGTT) group (n=4) open circles and experiment 2, control group (n=3) filled triangles, OGTT group (n=3) open triangles. Values are mean±SD. Dotted circles represent the end point in each experiment. SUV, standardized uptake value.

**Figure 3:**
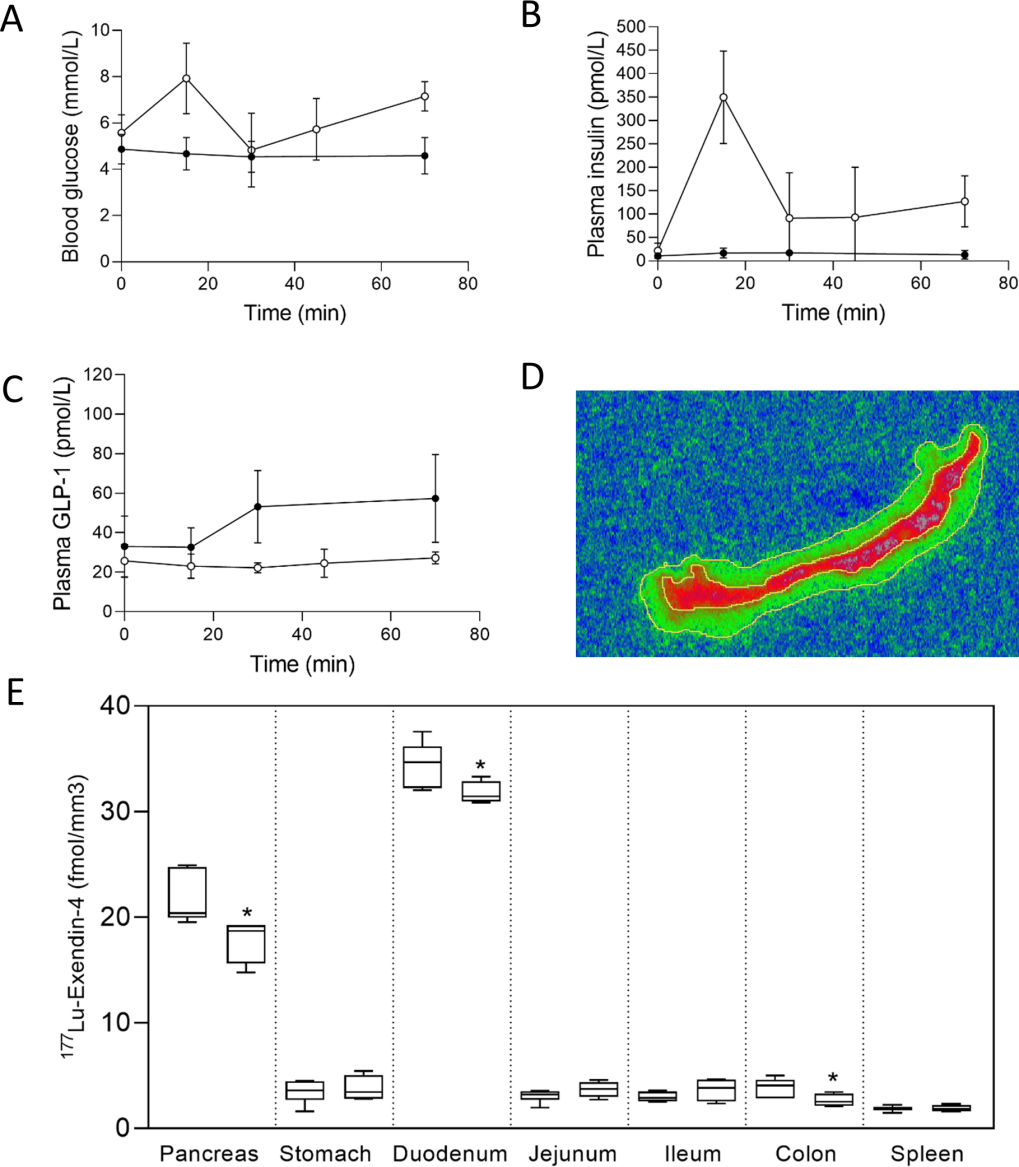
(A) Blood glucose, (B) plasma insulin and (C) plasma total glucagon-like peptide-1 (GLP-1) concentrations in pigs during experiment 1, control group (n=6) filled circles, oral glucose tolerance test (OGTT) group (n=4) open circles. Values are mean±SD. ^177^Lu-exendin-4 was injected at time=10 min. (D) Example of duodenum autoradiography image. Outer line represents entire section, inner line represents strong signal area. (E) ^177^Lu-exendin-4 in pancreas, stomach, duodenum (strong signal area), jejunum, ileum, colon and spleen measured by autoradiography in experiment 1 in 10 pigs, control (left, n=6) and OGTT (right, n=4). *indicates a significant (p<0.05) decrease in ^177^Lu-exendin-4 in the OGTT group compared with controls. Values are presented in box plots with first quartile, median and third quartile. Whiskers indicate min to max.

Autoradiography results from control pigs in experiment 1, injected with ^177^Lu-exendin-4, show the quantitative distribution of GLP-1Rs in pancreas, gastrointestinal tract and spleen (negative control) (figure 1). Autoradiography results from control pigs in experiment 2 showed similar GLP-1R distribution pattern as in experiment 1, although ^177^Lu-exendin-4 fmol/mm^3^ tissue was consistently slightly higher than in experiment 1 (figure 1), likely due to higher background in experiment 2 because of the earlier end point. ^177^Lu-exendin-4 background in blood was 2–3 times higher at the 30 min time-point in experiment 2, compared with the 60 min time-point in experiment 1 (figure 2A), which is in line with the increased binding in the negative control tissue (spleen), a reflection of the background binding. The plasma-to-whole blood ratio, that is, the proportion of ^177^Lu-exendin-4 available for tissue uptake, was similarly independent of treatment or experiment and therefore all binding data in the animals should be directly comparable (figure 2B). Hence, mean ^177^Lu-exendin-4, fmol/mm^3^ tissue presented below are data from experiment 1, which exhibited minimal influence from background binding. In the statistical comparison between spleen and low uptake gastrointestinal segments, data from experiment 2 were included in the analysis which was blocked for experiment to account for differences in background binding (for details see ‘Statistical analyses’ section). When the entire section was used to calculate uptake of ^177^Lu-exendin-4 (fmol/mm^3^ tissue), pancreas exhibited the highest uptake (21.65±2.46; figure 1). The uptake in duodenum was lower than in pancreas (18.29±1.84, paired t-test p=0.010). However, when only strong signal areas in duodenum (figure 3D) were compared with pancreas, uptake of ^177^Lu-exendin-4 was higher in duodenum (34.39±2.11, p<0.0001). Thus, there are areas in duodenum with higher GLP-1R density compared with pancreatic tissue. Low uptake of ^177^Lu-exendin-4 (fmol/mm^3^ tissue) was seen in the other gastrointestinal segments, but still higher than background as estimated by comparison with negative control tissue spleen (1.85±0.38), including stomach (3.45±1.08, p=0.0005), jejunum (3.06±0.60, p=0.0228), ileum (2.98±0.46, p=0.0156) and colon (3.88±0.91, p=0.0163).

### Location of GLP-1Rs

**Figure 4:**
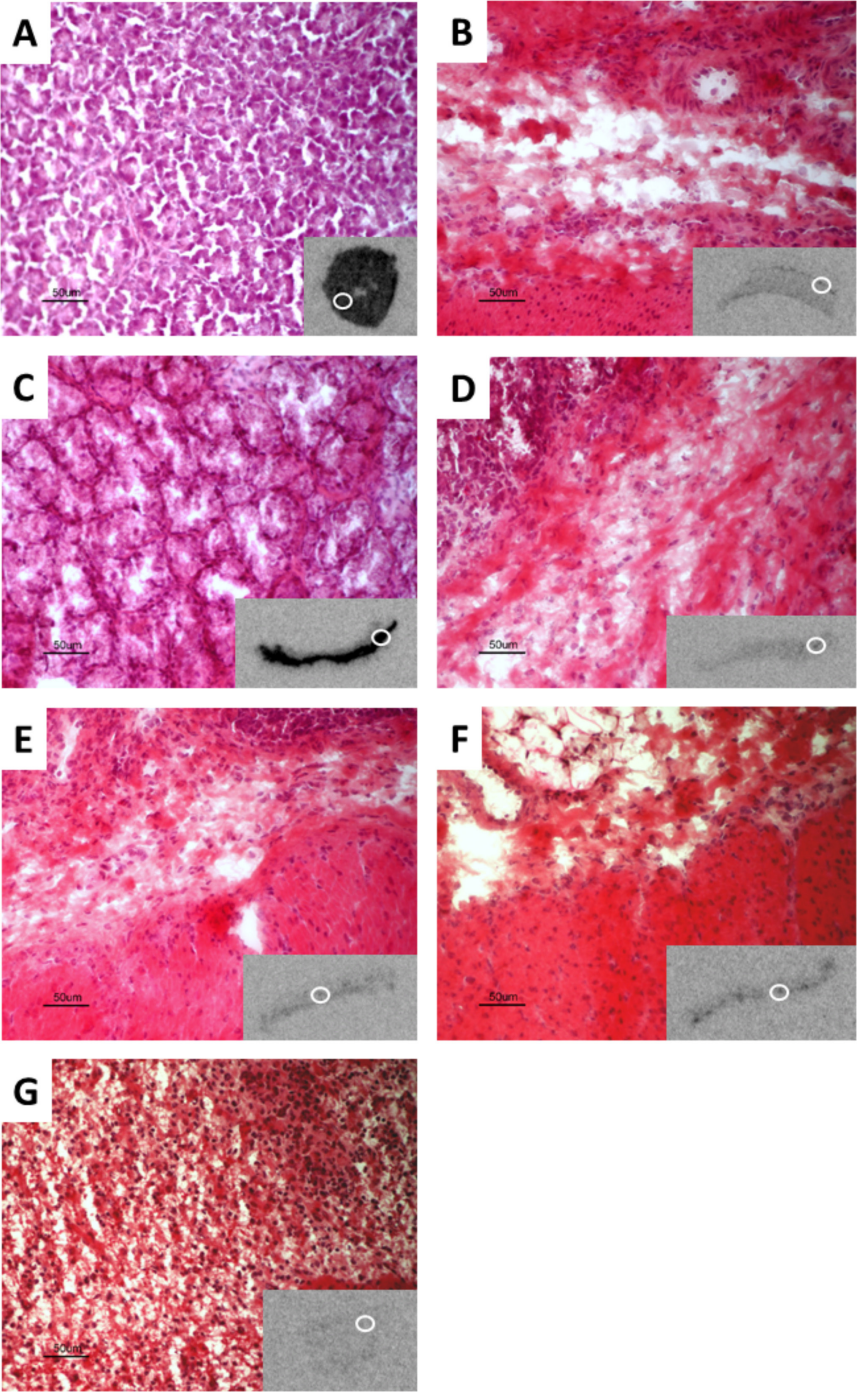
Example of autoradiography image and corresponding H&E-stained sections (12 µm, 20× magnification) from pancreas (A), stomach (B), duodenum (C), jejunum (D), ileum (E), colon (F) and spleen (G) from pigs injected with ^177^Lu-exendin-4. White circles on autoradiography images indicate magnified part of H&E-stained sections.

In experiment 1, autoradiography images were compared with H&E-stained sections to qualitatively assess the location of ^177^Lu-exendin-4 binding. Histology images are presented in figure 4. In pancreas, ^177^Lu-exendin-4 was distributed across the entire sections, with strong binding in both endocrine and exocrine tissue. In duodenum, the strong signal was located in the area corresponding to submucosa with Brunner’s glands. In the remaining gastrointestinal parts analyzed, the ^177^Lu-exendin-4 signal was relatively low and it was difficult to attribute it a specific anatomic structure with certainty. In the stomach, the ^177^Lu-exendin-4 signal seemed to be stronger in the muscularis mucosae and muscularis layer. In jejunum, ileum and colon, dispersed ^177^Lu-exendin-4 signal seemed to be located in submucosa and muscularis layer.

### GLP-1R occupancy during OGTT

During OGTT, in experiment 1, blood glucose concentrations and plasma insulin concentrations increased, while plasma concentrations of GLP-1 did not (figure 3A–C). The control group remained normoglycemic as expected. ^177^Lu-exendin-4/mm^3^ tissue was lower in pancreas (mean 17.6%, p=0.0355), duodenum (mean 7.9%, p=0.046) and colon (mean 31.8%, p=0.0481) in the OGTT group compared with controls (figure 3E). In experiment 2, there were no differences in ^177^Lu-exendin-4/mm^3^ tissue between OGTT group and controls.

### Correlation to PET/CT images in pigs

**Figure 5:**
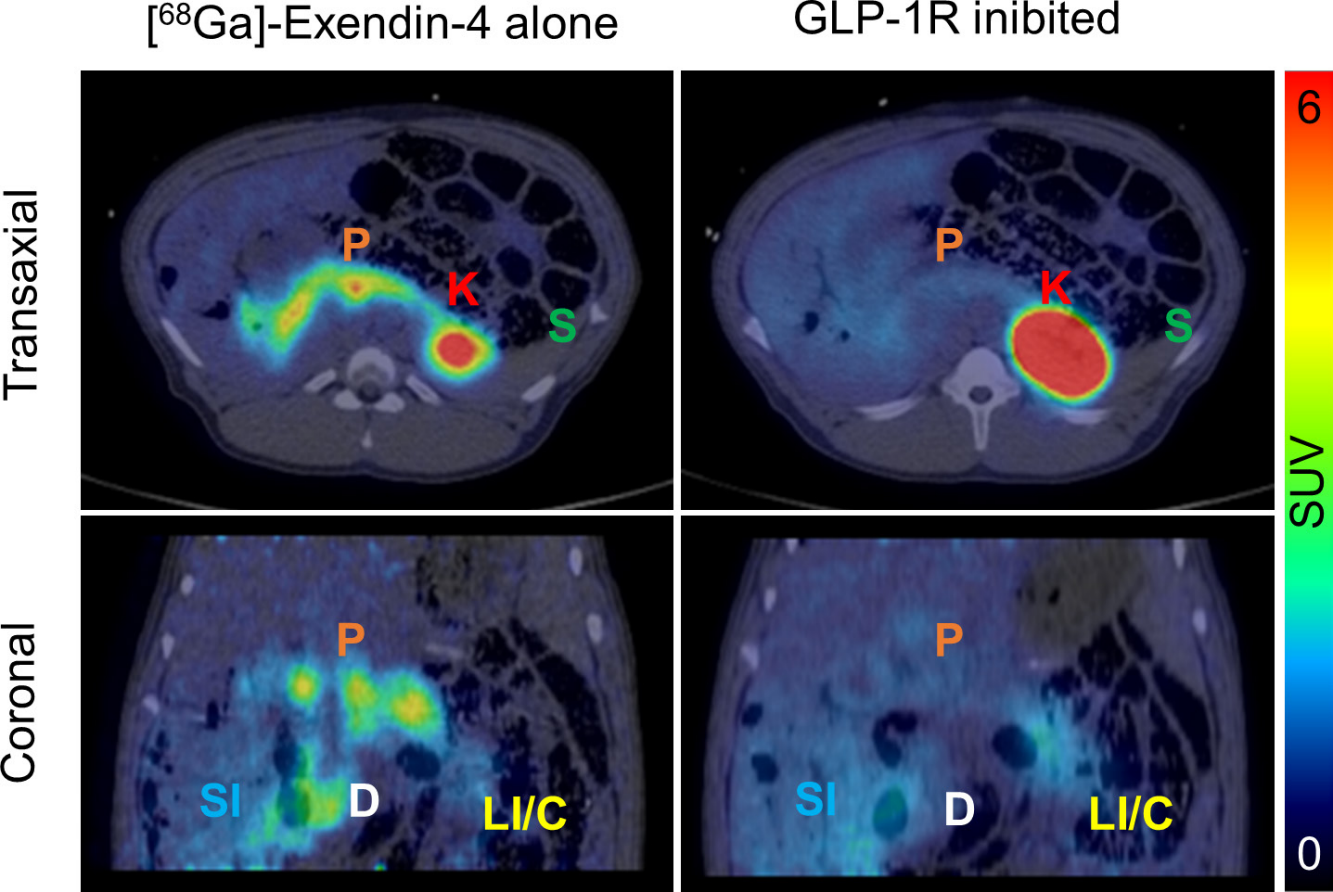
In vivo positron emission tomography/CT images after injection of ^68^Ga-exendin-4 in pigs. The examination in each pig was performed as baseline scans, that is, ^68^Ga-exendin-4 alone (left panels) or blocking scans, where unlabelled exendin-4 in excess was co-injected to occupy the glucagon-like peptide-1 receptor (GLP-1R) and inhibit the binding of ^68^Ga-exendin-4 (right panels). All images are normalized to standardized uptake value (SUV)=6 to enable direct comparison. Letters denote the location of pancreas (P), kidney (K), spleen (S), duodenum (D), small intestine (SI) and large intestine/colon (LI/C).

^177^Lu-exendin-4 ex vivo autoradiography revealed strong binding in pancreas and duodenum, with lesser binding in colon, followed by stomach and the small intestine and finally spleen. ^68^Ga-exendin-4 PET images, acquired in vivo, demonstrated a similar biodistribution with clear uptake in pancreas (figure 5, left panels). The pancreatic uptake was due to binding to the GLP-1R as it was inhibited by co-injection of around 150 µg unlabeled exendin-4 in excess (figure 5, right panels). There was high uptake in the kidney cortex and bladder, but this is well known to be the route of excretion of radiolabeled exendin-4^16^ and tubular uptake of exendin-4 in renal cortex is mediated through the scavenger receptors cubilin and megalin.^17^ Consequently, the uptake was uninfluenced by co-injection with unlabeled exendin-4 (figure 5, right panels). The only other tissue with clearly detectable binding, mediated by GLP-1R, was duodenum. No binding was seen in spleen. Diffuse binding was seen in small intestine and in the large intestines, but it was unclear whether it was influenced by administration of unlabeled exendin-4.

## Discussion

The present study revealed the quantitative distribution of the GLP-1R in porcine pancreas and gastrointestinal tract by ex vivo autoradiography. Two separate experiments confirmed that the method to assess target distribution of ^177^Lu-exendin-4 by ex vivo autoradiography in pigs is highly reproducible. The highest densities of GLP-1Rs were seen in pancreas and duodenum, which is in line with previous reports on human tissues examined with in vitro radiography^9^ and non-human primate tissues where highest IHC staining intensity was seen in pancreas and duodenum.^10^ In the present study, however, quantification of receptor binding in vivo was possible by ex vivo methods which objectively showed that there are areas in duodenum with higher receptor density than within the pancreas. In duodenum, there were confined areas with very strong radioactive signal in ex vivo autoradiography images, which made it easy to delineate only those areas during image processing. When compared with H&E-stained sections, the area with high ^177^Lu-exendin-4/mm^3^ tissue corresponded to submucosa with Brunner’s glands. In non-human primate and human duodenum, GLP-1Rs have been found to be numerous in Brunner’s gland epithelial cells,^9 10^ and this is a likely location also in the pig given the results from the present study. Brunner’s glands secretion protects the proximal duodenum from acid chyme^18^ and treatment with GLP-1 analogues in rodents increases production of substances involved in pathogen defence, barrier layer protection and mucosal healing.^19^

GLP-1 is well known to increase insulin secretion from beta-cells under hyperglycemic conditions in humans.^20^ The GLP-1–GLP-1R axis serves to temporarily increase insulin secretion in response to food intake. During OGTT, in the present experiment, a 17.6% decrease in binding of ^177^Lu-exendin-4 was observed, which we interpret to reflect occupancy of GLP-1Rs in the pancreas with endogenous GLP-1. The occupancy will depend on both the time of injection of ^177^Lu-exendin-4 in relation to glucose intake and to the experimental end point when tissues are collected for autoradiography. In experiment 1, a 10 min delay from oral glucose intake to ^177^Lu-exendin-4 was selected since intact GLP-1 in plasma (ie, recently secreted GLP-1) increase quickly and is significantly stimulated already 10 min after oral glucose intake in humans.^21^ Time is essential to remove unspecific signal from ^177^Lu-exendin-4 circulating in the bloodstream. In experiment 1, ^177^Lu-exendin-4 was allowed to circulate for 60 min, which minimized the background signal potentially caused by circulating ^177^Lu-exendin-4. However, the longer time to euthanasia the greater is the risk that GLP-1Rs, internalized by endogenous GLP-1, return to the cell surface and become available for ^177^Lu-exendin-4 to bind. Recirculation rate of GLP-1Rs in pigs is not known, but GLP-1Rs have been shown to return to the cell surface 45 min after being internalized by GLP-1 in mice.^22^ Based on the data from mice, some receptors initially internalized by endogenous GLP-1 in experiment 1 might have recirculated toward the end of the experiment, but this should have had minor effect since at that time ^177^Lu-exendin-4 concentration in plasma was low. Thus, the present experiment shows that only a small proportion of GLP-1 receptors is occupied by endogenous GLP-1 in response to an oral glucose load in young healthy pigs.

In the present experiment, the ^177^Lu-exendin-4 signal in the pancreas was equally distributed across the entire autoradiography image, which is in line with previous ex vivo autoradiography experiments where binding of exendin-4 in pig islets hardly differed from the exocrine tissue.^23^ In humans and non-human primates, GLP-1Rs are present in high density in beta-cells and lesser expression is seen in acinar cells within the pancreas.^9 10^ Other studies have described expression of the GLP-1R also in ductal cells,^24–26^ however these results could not be confirmed with more extensively validated methods.^9 10^ Porcine pancreatic uptake of ^68^Ga-exendin-4 measured by PET is not affected by streptozotocin-induced beta-cell ablation.^16^ Furthermore, high uptake of exendin-4 in porcine exocrine pancreas was also demonstrated recently in minipigs during fluorescence imaging.^27^ Hence, compared with humans, GLP-1Rs must be present in higher density in porcine exocrine tissue. Further experiments are needed to describe receptor localizations at the cellular level, but these results point to a different and species-specific role of the GLP-1–GLP-1R axis in the pancreas and glucose metabolism in pigs.

In contrast to the strong signal in well-confined areas in duodenum, the radioactive signal was more scattered in the other gastrointestinal segments, and it was not possible to further analyse those areas during image processing. When compared with spleen, the amount of ^177^Lu-exendin-4 was still higher in stomach, jejunum, ileum and colon, demonstrating the expression of GLP-1Rs in those tissues. Although it was difficult to assign ^177^Lu-exendin-4 signal with certainty to specific anatomic structures due to the relatively low resolution of the ex vivo autoradiography method, the signal seemed to be stronger in the muscularis mucosae and muscularis layer in the stomach. In non-human primate tissues, GLP-1Rs are present in muscles cells and parietal cells of the stomach.^10^ GLP-1 is known to decrease gastric motility and decrease gastric acid secretion.^28^ It is reasonable to assume that GLP-1Rs could be present in muscle cells also in the porcine stomach, which would explain the stronger signal areas. The GLP-1R has previously been located to myenteric plexus in the colon of humans and non-human primates^9 10^ and GLP-1 affects intestinal motility.^29^ Myenteric plexus neurons is a possible location also in the pig. GLP-1RAs treatment was recently shown to upregulate mRNA expression of proteins that ameliorated disease condition in a murine inflammatory bowel disease model and it was suggested that GLP-1RAs may affect gut homeostasis also in the distal parts of the gut.^19^ However, it remains to be elucidated if other cells, in addition to myenteric plexus neurons, in the colon also express the GLP-1R in mice and other species.

Interestingly, plasma GLP-1 concentrations did not increase in the present experiment despite higher oral glucose load than commonly given to humans, but consistently occupancy of the GLP-1Rs with endogenous GLP-1 was estimated as relatively low by the ex vivo ^177^Lu-exendin-4 assessment (eg, <20% in pancreas). This represents critical differences between pigs and humans. Porcine small intestines are relatively long, 30–40 times the length of the pig’s body,^30^ consequently it will take time for nutrients to reach L-cells of which there are few in the upper small intestine and plentiful in the distal part.^31 32^ Thus, since the human small intestines are shorter,^30^ the rapid increase in plasma concentrations of GLP-1 seen in humans after nutrient ingestion is likely to reflect early contact of glucose with proximal L-cells.^33^ Local infusion of glucose into the lumen of porcine ileum stimulates GLP-1 release,^34^ but perhaps GLP-1 is not an important incretin in pigs under physiological conditions. The present experiment also confirmed previous results that GLP-1Rs are equally distributed in the pancreas and not predominantly found in the islets,^16^ which is not the case for humans.^10^ Furthermore, when administering high doses of exendin-4 intravenously to pigs, severe tachycardia >200 bpm develops.^35^ Although exendin-4 increases heart rate in other species such as non-human primates, the tachycardia is not as severe.^35^ Pigs might not be as well adapted to high plasma concentrations of GLP-1 as other species. While pigs and humans share many physiological characteristics,^12^ and there are several advantages of using animal models in situations where experiments cannot be carried out in humans, differences in GLP-1 regulation and secretion must be kept in mind when using pigs as animal models.

The biodistribution of ^68^Ga-exendin-4 in pigs previously scanned by PET/CT demonstrated high similarity to the ex vivo autoradiography results. Pancreas and duodenum exhibited high binding with both methodologies, while spleen demonstrated negligible signal. The remainder of small intestine as well as the large intestine exhibited diffuse binding, also in line with the ex vivo autoradiography results. The discrepancy in sensitivity for the techniques is mainly due to the differences in spatial resolution (PET≈a few mm and ex vivo autoradiography≈100 µm). Thus, analogous studies investigating GLP-1R target distribution and occupancy can probably be performed in vivo using ^68^Ga-exendin-4, both in pigs and humans. In line with this, a recent ^68^Ga-exendin-4 PET study in humans demonstrated that a dual GLP-1/glucagon receptor agonist, given at doses which induced clinically relevant blood glucose-lowering effect in patients with type 2 DM, had an approximately 50% GLP-1R occupancy in pancreas.^36^ Interestingly, this dose also induced gastrointestinal side effects, highlighting the need for this kind of technology for understanding and fine-tuning the drug GLP-1R occupancy in different tissues for optimizing efficacy and reducing adverse events.

In conclusion, quantitative measures of GLP-1R distribution in porcine pancreas and gastrointestinal tract by ex vivo autoradiography revealed high density of GLP-1Rs in pancreas and duodenum, with areas in duodenum displaying the highest density. In pancreas, GLP-1Rs were equally distributed across the islets and the exocrine tissue. Low densities of GLP-1Rs were found in stomach, jejunum, ileum and colon. During OGTT, plasma concentrations of GLP-1 did not increase, and consequently only low and potentially clinically irrelevant GLP-1R occupancy by endogenous GLP-1 could be demonstrated in pancreas, duodenum and colon. This study demonstrates apparent differences in GLP-1 regulation and secretion between pigs and humans, which must be kept in mind when using pigs as animal model for diabetes. Since results from ex vivo autoradiography demonstrated high similarity to the biodistribution of ^68^Ga-exendin-4 in pigs previously scanned by PET, studies investigating GLP-1R target distribution and occupancy can be safely performed in vivo using ^68^Ga-exendin-4, both in pigs and humans.

## Data Availability

Data are available on reasonable request.
