# Supplementary material for: Exploring the GLP-1–GLP-1R axis in porcine pancreas and gastrointestinal tract in vivo by ex vivo autoradiography
Source: BMJ Open Diabetes Res Care. 2021 Apr 26;9(1):e002083. doi: 10.1136/bmjdrc-2020-002083 (PMC8076945; doi:10.1136/bmjdrc-2020-002083)
Supplement: Supplementary data [file bmjdrc-2020-002083supp001.pdf]

SUPPLEMENTARY DATA

Blood analyses prior to inclusion in experiment

To ensure that the pigs were healthy when enrolled in the experiment, blood samples were collected in EDTA- and serum-tubes after anaesthesia induction in connection with vein catheter surgery. EDTA preserved blood was analysed for complete blood cell counts including leucocyte differential counts, with an electronic cell counter validated for porcine blood (Advia 2120, Siemens, Erlangen, Germany). Serum samples were analysed for enzyme activities of aspartate amino transferase, alanine amino transferase, γ-glutamyltransferase, glutamate dehydrogenase and creatinine by automated equipment (Architect C4000, Abbott Diagnostics, North Ryde, Australia). Results from the blood analyses were within reference range for all pigs.

Indwelling vein catheters

Each pig was surgically fitted with an indwelling catheter in vena jugularis externa under aseptic conditions and general anaesthesia as previously described[1]. Procaine benzylpenicillin (Penovet® vet, 300 mg/mL; Boehringer Ingelheim Vetmedica, Ingelheim am Rhein, Germany) was administered preoperatively at a dose of 20 mg/kg BW intramuscularly and benzylpenicillin (Bensylpenicillin® Meda, 100 mg/mL; Meda, Solna, Sweden) was administered postoperatively twice daily for two consecutive days at a dose of 6 mg/kg BW i.v.

Preparation of [^{177}Lu]Lu-DOTA-Exendin-4-Nle14

Labelling of Nle^{14}-Lys^{40}(Ahx-DOTA)-Exendin-4 (TFA salt; DOTA-Exendin-Nle^{14}) with Lu-177 was accomplished using modified previously published method[2]. Lu-177 was purchased as hydrochloric solution (0.04 M, EndolucinBeta, 40 GBq/mL, ITG Isotope Technologies Garching

GmbH, Germany). DOTA-Exendin-4-Nle[14] (HGEGTFTSDLSKQ-Nle-EEEAVRLFIEWLKNGGPSSGAPPPSK(Ahx-DOTA)-NH2) was provided as trifluoroacetic acid (TFA) salt by Pi Chem, Austria. The labelling was conducted in an Eppendorf vial using 1 mL of 0.1 M HEPES (4-(2-hydroxyethyl)piperazine-1-ethanesulfonic acid) buffer to provide pH of 4.6-5.0 after addition of 60 µL (2560 MBq) of Lu-177. Thirty nanomoles of DOTA-Exendin-Nle[14] was added to the buffered Lu-177 solution and the reaction mixture was incubated for 30 minutes at 85°C. The crude extracts were purified using HLB solid phase extraction cartridges (Oasis, Waters) eluting the product with 60% ethanol buffered to pH 7.4. Thereafter ascorbic acid (200 mg) was added to the product and the latter was dispensed into three portions and kept in the freezer at -20°C for the subsequent use within 3 days. The stability in terms of radiochemical purity was controlled prior to the use in the animal experiments. The product was formulated in phosphate buffered saline for the administration to the animals. The radiochemical purity was analysed using analytical high-pressure liquid chromatography (HPLC) system from Beckman (Fullerton, CA) consisting of a 126 pump, a 166 UV detector, and a radiation detector coupled in series. The analysis was conducted using reversed-phase analytical column (Discovery BIO Wide Pore C5; 5cm x 4.6 mm) under the following conditions: A=10 mM TFA; B=70% acetonitrile (MeCN), 30% H_2O, 10mM TFA with UV-detection at 220 nm; linear gradient elution: 0–2 minutes at 35% B, 2-9 minutes at 35 to 100% B, 9-12 minutes at 100% B; flow rate was 2.0 mL/min. Data acquisition and handling was performed using the Beckman System Gold Nouveau Chromatography Software Package. The specific radioactivity of [^{177}Lu]-Exendin-4 was 100-120 MBq/nmol at the end of radiosynthesis.

<u>References</u>

1. Manell EAK, Ryden A, Hedenqvist P, et al. Insulin treatment of streptozotocin-induced diabetes re-establishes the patterns in carbohydrate, fat and amino acid metabolisms in growing pigs. *Lab Anim-Uk* 2014; 48: 261-269. DOI: Doi 10.1177/0023677213517683.

2. Velikyan I, Bulenga TN, Selvaraju R, et al. Dosimetry of [(177)Lu]-DO3A-VS-Cys(40)-Exendin-4 - impact on the feasibility of insulinoma internal radiotherapy. *American journal of nuclear medicine and molecular imaging* 2015; 5: 109-126. 2015/05/15.
